# Barriers Impact the Primary Healthcare Providers When Dealing With Emergency Cases: A Cross-Sectional Study in Al-Ahsa, Saudi Arabia

**DOI:** 10.7759/cureus.57344

**Published:** 2024-03-31

**Authors:** Ghufran AlAbdullah, Fatemah Al Ahmed, Zahra J Alatiyyah, Ghadeer Alibraheem, Abdullah Almuqahwi

**Affiliations:** 1 College of Medicine, King Faisal University, Al-Ahsa, SAU; 2 Family Medicine, King Faisal University, Al-Ahsa, SAU

**Keywords:** middle east, saudi arabia, attitude, knowledge, public health, physicians, emergency medicine, emergency medical services, primary healthcare

## Abstract

Introduction

Being the first in-line care providers, primary healthcare (PHC) physicians may encounter all forms of medical emergencies, ranging from minor complaints to major life‐threatening events; therefore, this study aims to assess the PHC physicians’ knowledge and attitude related to the diagnosis and management of emergency cases as well as their preferences for emergency medicine training courses and their preferred methods of training.

Methods

A descriptive cross-sectional study was conducted among physicians working at PHC in Al-Ahsa, Saudi Arabia, between November and December 2023, excluding those who do not work at PHC. The minimum required sample size was 192. A self-administered online questionnaire was developed using Google Forms and distributed conveniently through social media platforms. It consists of 22 items categorized into four sections: The first section focused on demographic information; the second section assessed knowledge related to the diagnosis and management of emergency cases commonly encountered in PHC, along with actual management experiences; the third section gauged physicians' attitudes, and the fourth section explored participants' preferences for emergency medicine courses and their preferred methods of training in this field. The Kruskal-Wallis rank sum and Wilcoxon rank sum tests were employed to identify predictors of knowledge and attitude.

Results

The study involved 193 participants, with 96 (50%) females and a median age of 30 years. The participants included 43 (22%) consultants, 69 (36%) family residents, 30 (16%) general practitioners, and 50 (26%) specialists. Participants reported a median of 4.0 years of experience in PHC in Saudi Arabia. The majority, 69 (36%), reported working in the hospital emergency department, with a reported median duration of three months. Roughly 84% of PHC physicians had a positive attitude toward emergency cases diagnosis and management, while 92% showed fair diagnostic knowledge of emergency cases, and 73% showed fair management knowledge. Higher knowledge in the diagnosis and management of PHC was linked to increasing age, being consultants, being employed in the emergency department, and having higher years of experience in PHC (p < 0.05). A positive attitude toward PHC was found to be associated with working as a consultant and being employed in the emergency department (p < 0.05). Approximately 133 (68.9%) expressed a preference for a course in wound care trauma, followed by central nervous system emergencies (n = 124, 64.2%), coronary artery disease emergencies (n = 116, 60.1%), and obstetrics/gynecologic emergencies (n = 114, 59.1%) with 160 (82.9%) favored training through practice in PHC under supervision of qualified staff.

Conclusion

While many PHC centers are well equipped and prepared for early stabilization and management of emergency cases, PHC physicians showed low competency in dealing with emergency cases. This indicates a great need for enhancing physician’s knowledge and skills regarding emergency situations. Nevertheless, courses like basic life support (BLS) and advanced trauma life support (ATLS) should be the bare minimum requirements for PHC physicians. Mote advance training and lectures should be organized to enhance PHC physician competencies to deal with different emergencies.

## Introduction

Primary health care (PHC) is defined by the World Health Organization (WHO) as essential health care made universally accessible to individuals and families in the community by means acceptable to them through their full participation and at a cost that both the community and the nation can afford. PHC includes services like family planning, maternity care, childcare, dental care, health educational services, communicable disease control, emergency care, and environmental health services [1]. It constitutes the cornerstone of the healthcare system, emphasizing a comprehensive societal approach to health that addresses individuals' needs across the continuum from health promotion to palliative care. The goal is to achieve the highest possible level of health and well-being with an emphasis on equitable distribution [2].

Aligned with Saudi Arabia's 2030 vision, the Health Sector Transformation Program (HSTP) endeavors to enhance healthcare quality, cost-effectiveness, and accessibility, prioritizing patient-centered care [3]. As the aging population, the complexities of chronic diseases, and the increasing specialization in health professions contribute to the fragmentation of care, there is a growing discourse on integrating emergency medical services (EMS) with PHC [4].

PHC physicians, being the initial responders to medical cases, confront a spectrum of symptoms, from minor ailments to life-threatening emergencies [5,6]. The broad scope of emergency situations challenges physicians to stay current and proficient in emergency medicine [7]. Unfortunately, there is a notable dearth of comprehensive literature locally and globally that explores and evaluates variables such as PHC physicians' knowledge, attitude, and practice (KAP) concerning emergency care [8].

International studies were carried out in Sri Lanka, Turkey, France, Asia, and the United States, demonstrating that PHC physicians’ attitudes and skill levels in emergency management in the PHC setting were inadequate [9-13]. In 2016, a study conducted in Egypt showed that most of the PHC visitors were unsatisfied with the emergency services rendered in terms of structure, process, and outcome [14].

Studies within the Kingdom of Saudi Arabia reveal a significant interest among PHC doctors in expanding their knowledge of emergency care [8]. Previous assessments in Dammam demonstrated variations in emergency medical services-related practice, learning needs, and preferred training methods among PHC physicians [15]. Notably, there was a recognized need for further training in specific areas, such as emergency cardiovascular and central nervous system management. A recent study in Taif highlighted the importance of training, revealing that physicians with longer work experience in PHC centers, those who completed an Advanced Cardiovascular Life Support (ACLS) course, and those with experience in emergency departments demonstrated better proficiency in dealing with emergencies [16].

Emergency medicine training was shown to be one of the most in-demand continuing medical education (CME) courses by researchers studying the need for CME among PHC providers, both nationally [13-15] and internationally [17,18].

To our knowledge, there is no published data evaluating the barriers impacting PHC physicians' ability to deal with emergency cases at the PHC level in Al-Ahsa, Saudi Arabia. The current study aims to comprehensively assess PHC physicians' knowledge, attitude, and practices related to the diagnosis and management of emergency cases presented to PHC, identify barriers, ascertain learning needs, and understand the preferred approaches.

## Materials and methods

Study design, setting, and population

This descriptive cross-sectional study was conducted among male and female physicians working at PHC in Al-Ahsa, Saudi Arabia, between November and December 2023, excluding physicians who do not work at PHC. Al-Ahsa contains 63 PHC centers serving 1,091,236 people representing 2.6% of the Saudi population with PHCs per 10,000 population ratio of 0.577 [19]. The sample size was calculated using the Raosoft calculator, considering a 95% confidence interval, 5% margin of error, and a total population of 380; the minimum required sample size was 192.

Data collection method

A self-administered online questionnaire was developed using Google Forms and distributed conveniently through various social media platforms specific to PHC (PHC) physicians in Al-Ahsa. A pilot study among 20 participants was conducted, and participants' feedback was used to improve clarity and understandability. The questionnaire was designed based on the available literature and validated by family medicine consultants [20]. It consists of 22 items categorized into four sections. The first section focused on demographic information, including age, gender, proficiency, place of graduation, years of experience in PHC, and work in a hospital emergency department. The second section assessed knowledge related to the diagnosis and management of emergency cases commonly encountered in PHC, along with actual management experiences. The third section gauged physicians' attitudes through five statements, each rated on a five-point Likert scale (1 = strongly disagree; 2 = disagree; 3 = not sure; 4 = agree; and 5 = strongly agree). The fourth section explored participants' preferences for emergency medicine training courses and their preferred methods of training in this field.

Data analysis approach

The collected data were entered and cleaned using an Excel spreadsheet. Subsequently, the information was transferred to the Statistical Package for Social Science Software (SPSS) program version 29 (IBM Corp., Armonk, NY) for analysis. Continuous variables were presented as mean and standard deviation, while categorical variables were expressed as numbers and percentages. Data normality was assessed using histograms and the Shapiro-Wilks test. For knowledge-related questions, participants aware of the diagnosis and management of emergency cases were coded as one, while unaware participants were coded as zero, and the total knowledge score was calculated. Those scoring ≥70% were considered to have fair knowledge. The attitude score was coded as (1 = strongly disagree; 2 = disagree; 3 = not sure; 4 = agree; and 5 = strongly agree), and individuals scoring ≥70% were considered to have a positive attitude. The Kruskal-Wallis rank sum and Wilcoxon rank sum tests were employed to identify predictors of knowledge and attitude, with a significance level set at p < 0.05.

Ethical considerations

Ethics for this research were approved by the King Faisal University Ethical Clearance Committee (KFU-REC-2023-NOV-ETHICS1572). The online questionnaire contained a consent form detailing the rights of the participants, including voluntary participation, confidentiality, anonymity, and a right to withdraw without justification.

## Results

The study involved 193 participants, with 96 (50%) females. Participants had a median age of 30 years (interquartile range [IQR]: 28-36). In terms of professional proficiency, the participants included 43 (22%) consultants, 69 (36%) family residents, 30 (16%) general practitioners, and 50 (26%) specialists.

The majority, 141 (73%), graduated from King Faisal University, while 44 (23%) graduated from other governmental Saudi medical colleges, five (2.6%) from international (non-Saudi) medical colleges, and three (1.6%) from other private Saudi medical colleges. Participants reported a median of 4.0 years of experience in PHC in Saudi Arabia. The majority, 69 (36%), reported working in the hospital emergency department, with a reported median duration of three months (IQR: 2-12 months) (Table 1).

**Table 1:** General characteristics of the participants n (%) *Median (IQR). IQR: Interquartile range.

Characteristic	N = 193
Sex	
Female	96 (50%)
Male	97 (50%)
Age (years)*	30 (28, 36)
Missing	4 (2.1%)
Proficiency	
Consultant	43 (22%)
Family medicine resident	69 (36%)
General practitioner	30 (16%)
Family medicine specialist	50 (26%)
Missing	1 (0.52%)
Place of graduation	
International (Non-Saudi) Medical College	5 (2.6%)
King Faisal University	141 (73%)
Other governmental Saudi medical college	44 (23%)
Other private Saudi medical college	3 (1.6%)
Years of experience in primary healthcare in Saudi Arabia*	4.0 (2.0, 8.0)
Working in hospital emergency department	
No	124 (64%)
Yes	69 (36%)
Duration of work in hospital emergency department (months)*	3 (2, 12)
Missing	126 (65.2%)

Acute heart failure, cardiac arrest, hypertension emergencies, asthma exacerbation, anaphylactic shock, acute abdomen, and cut wounds were noted to be encountered often by 34 (18%), 18 (9.3%), 121 (63%), 136 (70%), 13 (6.7%), 115 (60%), and 84 (44%) physicians, respectively. For sickle cell disease crises, participants often encountered them at 104 (54%), rarely at 62 (32%), and never at 27 (14%). Acute stroke was often encountered by 34 (18%), rarely by 77 (40%), and never by 82 (42%) of the participants. Epilepsy was often encountered by 24 (12%), rarely by 100 (52%), and never by 69 (36%) of the participants. Epistaxis was often reported by 109 (56%), rarely by 71 (37%), and never by 13 (6.7%) of the participants. Burns were often encountered by 64 (33%), rarely by 93 (48%), and never by 36 (19%). Hypoglycemia was often encountered by 141 (73%), rarely by 43 (22%), and never by 9 (4.7%) of the participants. The shock was often encountered by six (3.1%), rarely by 60 (31%), and never by 127 (66%) of the participants (Table 2).

**Table 2:** Primary healthcare physicians reported the emergency medicine cases they encountered n (%).

Characteristics	Never	Often	Rarely
Have you encountered any of the following diseases in your practice?			
Acute heart failure	67 (35%)	34 (18%)	92 (48%)
Cardiac arrest	94 (49%)	18 (9.3%)	81 (42%)
Hypertension emergency	4 (2.1%)	121 (63%)	68 (35%)
Asthma exacerbation	2 (1%)	136 (70%)	55 (28%)
Anaphylactic shock	88 (46%)	13 (6.7%)	92 (48%)
Acute abdomen	9 (4.7%)	115 (60%)	69 (36%)
Cut wound	13 (6.7%)	84 (44%)	96 (50%)
Sickle cell disease crisis	27 (14%)	104 (54%)	62 (32%)
Acute stroke	82 (42%)	34 (18%)	77 (40%)
Epilepsy	69 (36%)	24 (12%)	100 (52%)
Epistaxis	13 (6.7%)	109 (56%)	71 (37%)
Burn	36 (19%)	64 (33%)	93 (48%)
Hypoglycemia	9 (4.7%)	141 (73%)	43 (22%)
Shock	127 (66%)	6 (3.1%)	60 (31%)

Acute heart failure cases were managed most of the time by nine (4.7%), sometimes by 61 (32%), and never by 123 (64%) of the respondents. For cardiac arrest, 169 (88%) respondents reported never managing such cases, 21 (11%) managed them sometimes, and three (1.6%) managed them most of the time. Hypertension emergencies were managed by 59 (31%) most of the time, 99 (51%) sometimes, and 35 (18%) never, while asthma exacerbation was managed most of the time by 73 (38%), sometimes by 101 (52%), and never by 19 (9.8%).

Anaphylactic shock cases were managed most of the time by seven (3.6%), sometimes by 57 (30%), and never by 129 (67%); however, acute abdomen cases were managed most of the time by 57 (30%), sometimes by 87 (45%), and never by 49 (25%). Cut wounds were managed most of the time by 23 (12%), sometimes by 113 (59%), and never by 57 (30%). Sickle cell disease crises were managed most of the time by 66 (34%), sometimes by 52 (27%), and never by 75 (39%). Acute stroke cases were managed most of the time by 10 (5.2%), sometimes by 38 (20%), and never by 145 (75%). Epilepsy cases were managed most of the time by six (3.1%), sometimes by 75 (39%), and never by 112 (58%). Epistaxis cases were managed most of the time by 55 (28%), sometimes by 117 (61%), and never by 21 (11%). Burns were managed most of the time by 25 (13%), sometimes by 89 (46%), and never by 79 (41%). Hypoglycemia cases were managed most of the time by 102 (53%), sometimes by 70 (36%), and never by 21 (11%). Shock cases were managed most of the time by three (1.6%), sometimes by 20 (10%), and never by 170 (88%) (Table 3).

**Table 3:** Primary healthcare physicians reported the management of emergency medicine cases they encountered n (%). PHC: Primary healthcare.

Characteristics	Most of the time	Sometimes	Never
Have you actually managed these cases in your PHC?			
Acute heart failure	9 (4.7%)	61 (32%)	123 (64%)
Cardiac arrest	3 (1.6%)	21 (11%)	169 (88%)
Hypertension emergency	59 (31%)	99 (51%)	35 (18%)
Asthma exacerbation	73 (38%)	101 (52%)	19 (9.8%)
Anaphylactic shock	7 (3.6%)	57 (30%)	129 (67%)
Acute abdomen	57 (30%)	87 (45%)	49 (25%)
Cut wound	23 (12%)	113 (59%)	57 (30%)
Sickle cell crisis	66 (34%)	52 (27%)	75 (39%)
Acute stroke	10 (5.2%)	38 (20%)	145 (75%)
Epilepsy	6 (3.1%)	75 (39%)	112 (58%)
Epistaxis	55 (28%)	117 (61%)	21 (11%)
Burn	25 (13%)	89 (46%)	79 (41%)
Hypoglycemia	102 (53%)	70 (36%)	21 (11%)
Shock	3 (1.6%)	20 (10%)	170 (88%)

Higher knowledge in the diagnosis and management of PHC was linked to increased age (p < 0.001). Consultants (p < 0.001) and individuals employed in the emergency department (p = 0.03) demonstrated elevated knowledge in diagnosing and managing PHC conditions. Additionally, a greater number of years of experience in PHC is associated with increased knowledge in the diagnosis and management of PHC (p < 0.001) (Table 4).

**Table 4:** Determinants of management and diagnostic knowledge of the emergency cases among primary healthcare physicians ^1 ^Knowledge score: Median (IQR). ^2 ^Kruskal-Wallis rank sum test; Wilcoxon rank sum test. IQR: Interquartile range.

Characteristics	N = 193^1^	p-value^2^
Age (years)		<0.001
24-34	26.0 (21.0, 28.0)	
35 years and above	28.0 (27.0, 28.0)	
Sex		>0.9
Female	27.0 (21.0, 28.0)	
Male	26.0 (23.0, 28.0)	
Proficiency		<0.001
Consultant	28.0 (27.0, 28.0)	
Family resident	24.0 (21.0, 28.0)	
General practitioner (GP)	24.0 (16.3, 28.0)	
Specialist	27.5 (26.0, 28.0)	
Working in hospital emergency department		0.030
No	26.0 (21.0, 28.0)	
Yes	28.0 (24.0, 28.0)	
Duration of work in hospital emergency department (months)		>0.9
0-5 months	27.0 (24.0, 28.0)	
6 months and above	28.0 (23.0, 28.0)	
Years of experience in primary healthcare in Saudi Arabia		<0.001
0-5 years	25.0 (20.0, 28.0)	
6 years and above	28.0 (26.0, 28.0)	

A positive attitude toward PHC was found to be associated with working as a consultant (p = 0.017) and being employed in the emergency department (p < 0.001) (Table 5).

**Table 5:** Determinants of the attitude of the emergency cases among primary healthcare physicians ^1^ Attitude score: Median (IQR). ^2^ Kruskal-Wallis rank sum test; Wilcoxon rank sum test. IQR: Interquartile range.

Characteristics	N = 193^1^	p-value^2^
Age (years)		0.11
24-34	16.0 (14.0, 17.0)	
35 years and above	16.5 (14.0, 19.0)	
Sex		0.5
Female	16.0 (14.0, 18.0)	
Male	16.0 (14.0, 18.0)	
Proficiency		0.017
Consultant	17.0 (14.0, 19.0)	
Family resident	16.0 (14.0, 17.0)	
General practitioner (GP)	15.0 (13.0, 16.0)	
Specialist	16.5 (14.0, 18.0)	
Working in hospital emergency department		<0.001
No	15.0 (13.8, 17.0)	
Yes	17.0 (15.0, 19.0)	
Duration of work in hospital emergency department (months)		0.2
0-5 months	17.0 (14.0, 18.8)	
6 months and above	17.0 (16.0, 19.0)	
Years of experience in primary healthcare in Saudi Arabia		0.10
0-5 years	16.0 (14.0, 17.0)	
6 years and above	16.0 (14.0, 19.0)	

**Figure 1:**
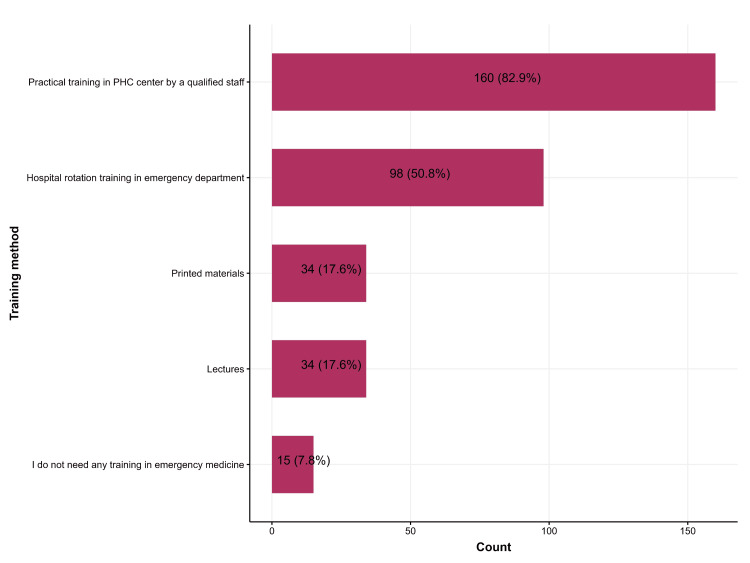
The preferred method of emergency medicine training reported by primary healthcare physicians PHC: Primary healthcare.

Figure 1 shows the preferred training choices reported by PHC physicians. About 160 (82.9%) favored practical training in PHC conducted by qualified staff, 98 (50.8%) preferred hospital rotation training, 34 (17.6%) expressed a preference for printed materials, another 34 (17.6%) for lectures, and 15 (7.8%) indicated that they did not desire any training (Figure 1).

**Figure 2:**
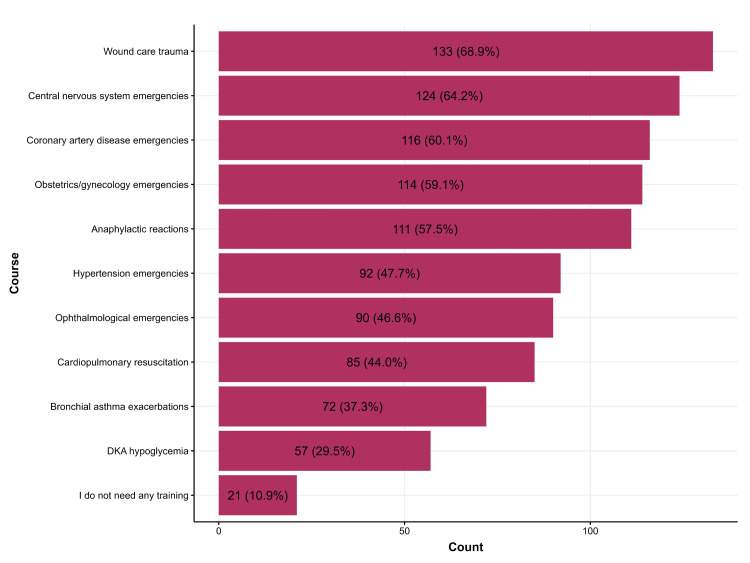
Primary healthcare physicians' preferences for training courses in emergency medicine DKA: Diabetic ketoacidosis.

Figure 2 shows the PHC physicians' preferences for specific courses. The largest proportion of participants, 133 (68.9%), expressed a preference for a course in wound care trauma, followed by central nervous system emergencies (n = 124, 64.2%), coronary artery disease emergencies (n = 116,60.1%), and obstetrics/gynecologic emergencies (n = 114,59.1%) (Figure 2).

**Figure 3:**
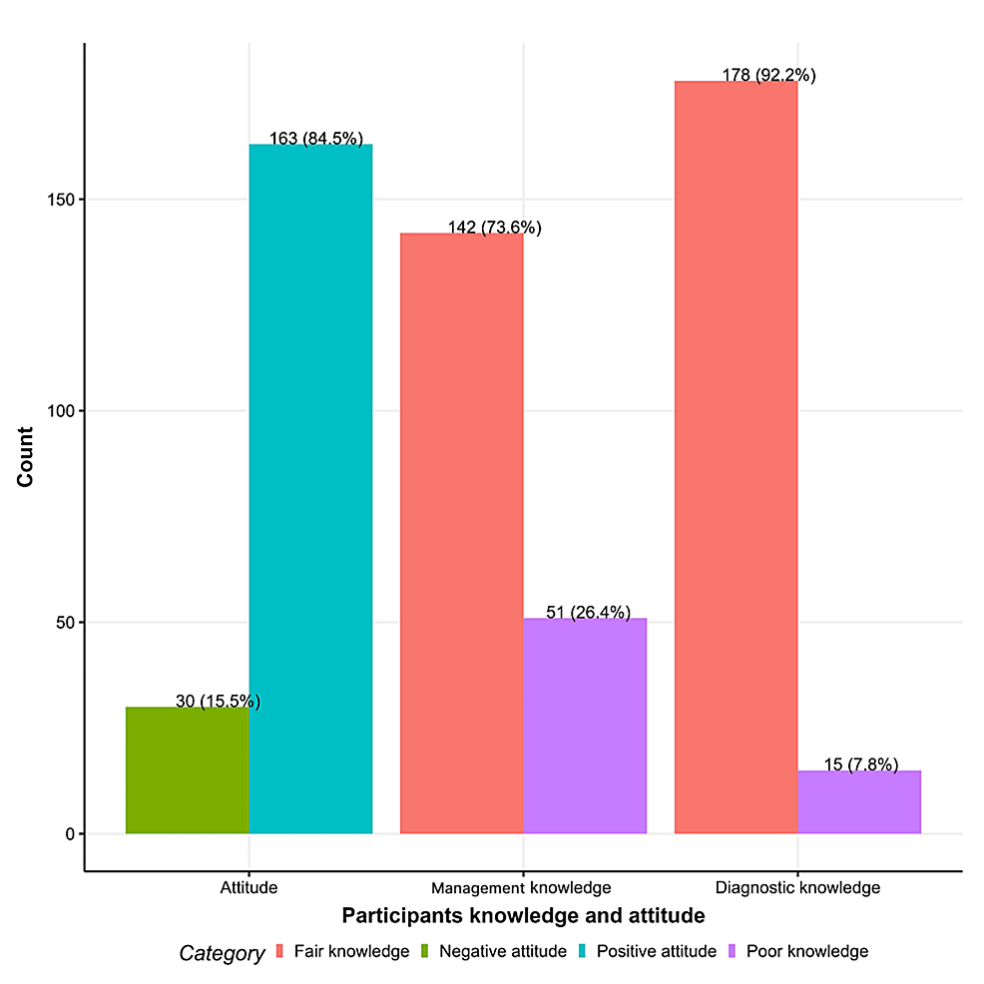
Knowledge and attitude among primary healthcare physicians regarding emergency cases presented to primary healthcare

Figure 3 shows the knowledge and attitude among PHC physicians regarding emergency cases presented to PHC. Approximately 142 (73.6%) and 178 (92.2%) had a fair management and diagnostic knowledge of PHC, while 163 (84.5%) had a positive attitude (Figure 3).

## Discussion

This study aimed to evaluate the emergency medicine practices of PHC physicians in relation to EMS. Male to female ratio in this study is about 1:1, 36% were family physicians, and 26% were specialists. About 73% graduated from King Faisal University, and 23% were from other governmental Saudi medical colleges. About 35% and 48% either never or rarely encountered emergencies like acute heart failure in their practice at the PHC levels. It is possible that these cases correctly go directly to emergency departments, or such cases do present to PHC but were not correctly identified by PHC physicians. About 64% of PHC physicians admitted that they never managed acute heart failure at their centers. The picture is similar to other types of emergencies including cardiac arrest, acute abdomen, epilepsy, hypoglycemia, and shock. PHC physicians admitted never or rarely encountered or managed these cases at the PHC levels.

Participants demonstrated an overall positive attitude (84%), with 92% showing fair diagnostic knowledge of emergency cases and 73% showing fair management knowledge. When investigating determinants of management and diagnostic knowledge of PHC physicians, most of the studied factors turned out to be non-statistically significant, apart from being above 35 years old (p < 0.001), having consultant and specialist level of proficiency (p < 0.001), working in hospital emergency department (0.030), and having six plus years of experience in PHC in Saudi Arabia (p < 0.001). Determinants of the attitude of PHC physicians include proficiency - consultant level (p = 0.017), place of graduation (p < 0.001), and working in the hospital emergency department (p < 0.001).

A study conducted in Al Madinah, Saudi Arabia, revealed that 39% of physicians demonstrated a poor level of competency in emergency case identification and management. Males were better than females in dealing with emergency cases (p = 0.021), and physicians who attended BLS and ATLS courses were more competent than their colleagues who did not attend these courses. PHC physicians admitted that insufficient knowledge and skills regarding emergency case management is a major barrier for them to deal with emergency situations [21]. In Jeddah (Eastern Province, Saudi Arabia), 97% attended BLS courses, yet 83% did not attend ATLS courses. About 72% of PHC physicians in Jeddah reported previous experience in working in emergency departments. Older physicians, those who worked more than five years in PHCs, and non-Saudi nationalities demonstrated higher competencies in emergency case management [22].

In the Dammam area, 87% of PHC physicians had good diagnostic knowledge, and 47% had a good management score for emergency cases. Nevertheless, 63% had a fair attitude toward emergency management services. The most commonly encountered emergencies in PHC include bronchial asthma, cut wounds, and burns [15]. In Riyadh, those who showed low competence in dealing with emergency cases were middle-aged physicians, females, Saudi, and those who did not report working in emergency units; the same findings were reported by Alaa et al. at Taif, Saudi Arabia [23]. In Spain, the most commonly encountered barrier to emergency case management by PHC physicians was a lack of practical skills in dealing with emergency cases [24].

However, many studies called for better enhancing emergency case management at the PHC levels, thus decreasing the load on the emergency department and helping in stabilization and early initiation of diagnosis and management of emergency cases. It is also recommended that more integration between PHC and emergency departments should be planned [4,7,25,26].

This study has some limitations, including the small, yet representative sample size. It would better allow more generalization abilities if more participants from different geographical regions were included. Also, the self-rating nature of our data could lead to an overestimation of competence among PHC physicians.

## Conclusions

This cross-sectional study aimed to evaluate the emergency medicine practices of PHC physicians and included 193 participants. About 36% were family residents, and only about a third of the study participants worked in hospital emergency departments; 84% of PHC physicians had a positive attitude toward emergency cases diagnosis and management; 92% showed fair diagnostic knowledge of emergency cases, and 73% showed fair management knowledge. While many PHC centers are well equipped and prepared for early stabilization and management of emergency cases, PHC physicians showed low competency in dealing with emergency cases. This indicates a great need for enhancing physician’s knowledge and skills regarding emergency situations. Nevertheless, courses like BLS and ATLS should be the bare minimum requirements for PHC physicians. Mote advance training and lectures should be organized to enhance PHC physician competencies to deal with different emergencies.
